# Complete sequence of *Cynanchum rostellatum* (Apocynaceae: Asclepiadoideae) chloroplast genome and its phylogenetic analysis

**DOI:** 10.1080/23802359.2022.2102444

**Published:** 2022-07-29

**Authors:** Lixin Pei, Shengnan Shu, Baoyu Ji, Ning Cui

**Affiliations:** a College of Pharmacy, Henan University of Chinese Medicine, Zhengzhou, China; b Central Laboratory, Shandong Academy of Chinese Medicine, Ji’nan, China; c School of Pharmacy and Chemical Engineering, Zhengzhou University of Industry Technology, Zhengzhou, China

**Keywords:** *Cynanchum rostellatum*, chloroplast genome, phylogeny

## Abstract

*Cynanchum rostellatum* (Turcz.) Liede and Khanum 2016 is a perennial herbaceous twining vine that is widely distributed in Japan, South Korea, the United States of America, and China. In this study, the complete chloroplast (cp) genome of *C. rostellatum* was sequenced using the Illumina platform and assembled for the first time. This plastome has a circular structure with a length of 160,641 bp. The GC content of the plastome was 37.82%. The cp genome contained 113 unique genes, including 79 protein-coding, 30 transfer RNA, and four ribosomal RNA genes. Phylogenetic analysis based on the complete cp genome sequences of the Asclepiadoideae subfamily showed that *C. rostellatum* was closely related to *C. bungei* in the genus *Cynanchum*. These results provide useful information for both phylogenetic research and the utilization of *C. rostellatum*.

*Cynanchum rostellatum* (Turcz.) Liede and Khanum 2016 is a perennial herbaceous twining vine of the Asclepiadoideae subfamily of Apocynaceae, with the heterotypic synonym *Metaplexis japonica* in the *Flora Reipublicae Popularis Sinicae* database (http://www.iplant.cn/; Ma and Clemants 2006). The herb contains milky latex and is widely distributed in Japan, South Korea, the United States of America, and China (Welsh and Anderson 1962). The fiber of *C. rostellatum* has considerable industrial application prospects, and the fruit, root, stem, leaf, seed hair, and milky juice of *C. rostellatum* have a long history in traditional Chinese medicine (Yang et al. 2006; Wang et al. 2020). The chemical components of *C. rostellatum* have been shown to have a variety of pharmacological activities, such as antitumor, antioxidant, antibacterial, immunosuppressive, and neuroprotective activities (Zhang et al. 2014; Wei et al. 2019). Although *C. rostellatum* has significant economic and medicinal value, genetic and evolutionary research is extremely rare. In this study, we report the complete chloroplast (cp) genome of *C. rostellatum* and examine its phylogenetic position within the subfamily Asclepiadoideae. It is expected to lay the foundation for further molecular studies and the utilization of this species.

Fresh leaves of *C. rostellatum* were collected from Xixia County, Nanyang City, Henan Province (33°38′N, 111°43′E). The specimen and DNA were deposited at the Herbarium of Henan University of Traditional Chinese Medicine, Henan, China (contact person: Lixin Pei, xlpxlp@aliyun.com), under voucher number HNPS2020-12-032. Total genomic DNA was extracted using a Dneasy Plant Mini Kit (Qiagen, CA, USA) according to the manufacturer’s instructions (Cui et al. 2020). The genomic library for 150 bp paired-end sequencing was produced using the Illumina HiSeq 1500 platform (Illumina Inc., USA). The cp genome of *C. auriculatum* (NC029460) was used as a reference for assembly and annotation. The complete chloroplast genome was assembled using Getorganelle (v. 1.1.1; Jin et al. 2020), and annotated by Plann (Huang and Cronk 2015). The novel cp genome was submitted to the NCBI database (www.ncbi.nlm.nih.gov) under the GenBank accession number OL689165.

The cp genome of *C. rostellatum* contained circular double-stranded DNA and displayed a typical quadripartite structure, including two inverted repeat (IR) regions with lengths of 23,841 bp and 23,836 bp, separated by a large single-copy (LSC) region of 92,051 bp and a small single copy (SSC) region of 20,913 bp. The GC content of the plastome was 37.82%. The genome contained a set of 132 genes, of which 113 were unique and 19 were duplicated. A total of 87 protein-coding genes (79 unique genes) were annotated and were mainly involved in processes related to photosynthesis and gene expression. Eleven protein-coding genes (*rps16, atpF, rpoC1, petB, petD, rpl16*, two *rpl2* genes, two *ndhB* genes, and *ndhA*) and eight tRNA genes (*trnK-UUU, trnG-UCC, trnL-UAA*, *trnV-UAC, trnI-GAU, trnA-UGC, trnA-UGC,* and *trnI-GAU*) contained one intron, and two genes (*clpP* and *ycf3*) contained two introns.

To elucidate the evolutionary relationship of *C. rostellatum*, the cp genomes of 21 Asclepiadoideae species were downloaded from the NCBI GenBank database. We aligned the plastomes using MAFFT and constructed a maximum likelihood (ML) tree (Figure 1) using RAxML (v.8.2.9), using the GTRGAMMA model with 1000 rapid bootstrap replicates (Cui et al. 2020). Our plastome phylogeny showed that *C. rostellatum* is most closely related to *C. bungei* in the genus *Cynanchum*. *Cynanchum rostellatum* and seven *Cynanchum* species were clustered into one branch indicating that *C. rostellatum* was likely placed in the *Cynanchum* genus, and not in *Metaplexis*, during taxonomic classification. In conclusion, the cp genome of *C. rostellatum* provides a theoretical basis for a better understanding of the evolutionary patterns and for improving its taxonomic classification.

**Figure 1.**
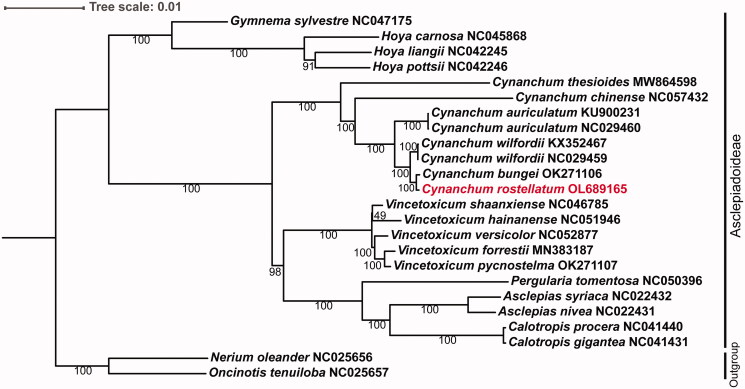
Phylogenetic tree constructed by using the whole chloroplast genome sequences of Asclepiadoideae subfamily with the maximum likelihood method. Numbers near each branch are the bootstrap values.

## Data Availability

The complete chloroplast genome of *Cynanchum rostellatum* assembled here is available in the GenBank of NCBI (https://www.ncbi.nlm.nih.gov/genbank, accession no. OL689165). The associated BioProject, BioSample and SRA numbers are PRJNA785091, SAMN23527168 and SRR17082022, respectively.
